# Ab Initio Modeling of the Herpesvirus VP26 Core Domain Assessed by CryoEM Density

**DOI:** 10.1371/journal.pcbi.0020146

**Published:** 2006-10-27

**Authors:** Matthew L Baker, Wen Jiang, William J Wedemeyer, Frazer J Rixon, David Baker, Wah Chiu

**Affiliations:** 1 National Center for Macromolecular Imaging, Verna and Marrs McLean Department of Biochemistry and Molecular Biology, Baylor College of Medicine, Houston, Texas, United States of America; 2 Department of Biological Sciences, Purdue University, West Lafayette, Indiana, United States of America; 3 Department of Biochemistry, Michigan State University, East Lansing, Michigan, United States of America; 4 MRC Virology Unit, Institute of Virology, Glasgow, United Kingdom; 5 Department of Biochemistry, University of Washington, Seattle, Washington, United States of America; University of California San Diego, California

## Abstract

Efforts in structural biology have targeted the systematic determination of all protein structures through experimental determination or modeling. In recent years, 3-D electron cryomicroscopy (cryoEM) has assumed an increasingly important role in determining the structures of these large macromolecular assemblies to intermediate resolutions (6–10 Å). While these structures provide a snapshot of the assembly and its components in well-defined functional states, the resolution limits the ability to build accurate structural models. In contrast, sequence-based modeling techniques are capable of producing relatively robust structural models for isolated proteins or domains. In this work, we developed and applied a hybrid modeling approach, utilizing cryoEM density and ab initio modeling to produce a structural model for the core domain of a herpesvirus structural protein, VP26. Specifically, this method, first tested on simulated data, utilizes the cryoEM density map as a geometrical constraint in identifying the most native-like models from a gallery of models generated by ab initio modeling. The resulting model for the core domain of VP26, based on the 8.5-Å resolution herpes simplex virus type 1 (HSV-1) capsid cryoEM structure and mutational data, exhibited a novel fold. Additionally, the core domain of VP26 appeared to have a complementary interface to the known upper-domain structure of VP5, its cognate binding partner. While this new model provides for a better understanding of the assembly and interactions of VP26 in HSV-1, the approach itself may have broader applications in modeling the components of large macromolecular assemblies.

## Synopsis

Efforts in structural genomics have targeted the systematic determination of all protein structures primarily using X-ray crystallography and nuclear magnetic resonance. These initiatives have typically focused on domains, single-protein and in some cases small complexes, and as such macromolecular machines are relatively underrepresented. However, in recent years, electron cryomicroscopy (cryoEM) has assumed an increasingly important role in determining the structure of large macromolecular machines in their biologically active states to intermediate resolutions (5–10 Å). Concurrently, modeling techniques, such as comparative and ab initio modeling, have played an increasingly important role in structure determination of small proteins not amenable to other structural techniques. In this work, Baker and colleagues have leveraged ab initio modeling and cryoEM to assess and identify structural models for the macromolecular components within a large complex. Specifically, the cryoEM density can be used to select the most native-like models from a large gallery of potential models. Applied to the smallest herpesvirus capsid protein, VP26 (12 kDa), it was possible to determine its core domain structure (residues 42–105), which helped to elucidate interactions among the structural protein in the virion. Beyond VP26, these techniques potentially provide a new pathway for accurate structure determination of proteins in their biological and functional states.

## Introduction

Multicomponent macromolecular assemblies contribute to nearly all essential biological processes, and as such, the analysis of these assemblies is critical in understanding basic cell biology and is potentially relevant to treating disease. Structural biology offers the promise of understanding biological molecules through the study of their architecture and shape. Traditionally, such understanding has been acquired by determining the 3-D structures of individual proteins or small complexes using X-ray crystallography and nuclear magnetic resonance spectroscopy. In recent years, electron cryomicroscopy (cryoEM) has become increasingly important in determining structures of complex macromolecular assemblies, including the ribosome [1], acrosomal bundle [2], bacterial flagella [3], clathrin [4], and viruses [5]. However, at present, the best cryoEM density maps are still limited to intermediate resolutions (6–10 Å), making it a daunting challenge to resolve high-resolution structural features. While there are examples of computational tools for detecting structural features [6–8] and for fitting the components of an assembly at intermediate resolutions [9–14], methods are just beginning to emerge for generating models in the context of cryoEM density maps [15].

### Protein Modeling

In many cases, it is not possible to directly solve the structure of a protein using experimental techniques. For these proteins, modeling represents the only effective way of determining the structure. In general, modeling can be divided into two categories; template modeling (threading, comparative modeling) and template-free or ab initio modeling [16]. While the approach of these techniques varies, the overall concept for both involves applying a variety of constraints to optimally produce a structural model that is useful in understanding the overall structural characteristics of the protein even in the most difficult modeling cases.

As the name suggests, template-based modeling requires a related structure for the modeling of a target protein sequence. In these types of modeling cases, it is possible to generate a set of models based on single, multiple, or even alternate templates and alignments, from which the best model in a set is selected through statistical analysis [17]. Recent work has shown that cryoEM density can discriminate between alternative models [14]. This has led to a sequence alignment, modeling, and refinement protocol that utilizes both cryoEM density and comparative modeling considerations in an iterative comparative modeling procedure [15].

Unlike comparative modeling, ab initio modeling does not rely on a template structure, rather it focuses on the physics of protein folding. In general, ab initio methods are restricted to relatively small proteins and are not as good as models based on related structural templates. Most ab initio modeling can be thought of as an optimization problem attempting to identify the native structure of an individual protein by finding the lowest energy model from a large gallery of possible models. To date, Rosetta [18] has been shown to be relatively successful in ab initio modeling. Rosetta is based on a picture of protein folding in which local sequence segments rapidly alternate between different possible local structures, and folding occurs when the conformations and relative orientations of these local segments combine to form low-energy global structures. The distribution of conformations sampled by an isolated chain segment is approximated by the distribution of conformations adopted by that sequence segment and related sequence segments in the protein structure database. Nonlocal interactions are optimized by a Monte Carlo search through the set of conformations that can be built from these fragment ensembles; resulting structures therefore have low free energy for local and nonlocal interactions.

### Herpes Simplex Virus Type-1

*Herpesviridae* is a family of large DNA viruses, several of which infect humans and cause diseases such as chicken pox, mononucleosis, and facial and genital lesions [19]. Some members of the family are highly pervasive; herpes simplex virus type-1 (HSV-1), the prototypical member of the family, is present in 40%–80% of the worldwide population [20].

One of the largest and most complex human viruses, HSV-1 (~2000 Å in diameter) is composed of an outer glycoprotein-containing envelope surrounding an amorphous protein layer, the tegument, which in turn surrounds an icosahedral nucleocapsid containing the linear dsDNA genome [21]. The HSV-1 capsid is arranged on a T = 16 icosahedral lattice and consists of 150 hexons and 11 pentons connected by 320 heterotrimeric complexes known as triplexes. Both pentons and hexons are composed of VP5, the major capsid protein (149 kDa). Present in the hexon subunits but absent from pentons is another protein, VP26 (12 kDa), which forms a hexameric ring on the outermost surface of each hexon [22]. The triplexes, containing one copy of VP19C (50 kDa) and two copies of VP23 (34 kDa), bridge the neighboring hexons and pentons [23]. These proteins will spontaneously self-assemble to form capsids in the presence of an internal scaffolding protein that is lost during DNA packaging [24,25].

CryoEM and structural analysis of the capsid have produced a structural model for the architecture/topology of VP5 [26]. The X-ray structure of a bacterially expressed domain of VP5 comprising residues 451-1054 was localized to the upper domain of the hexon and penton subunits extracted from the 8.5-Å cryoEM map of the HSV-1 capsid [27]. Additional structural analysis has resulted in a topological model for the remaining portion of VP5, revealing a structural similarity, and possible evolutionary relationship, to the capsid protein in tailed dsDNA bacteriophages [28].

### HSV-1 VP26

While all mammalian and avian herpesviruses encode for a small capsid protein that binds to the major capsid protein (VP26 and VP5 in HSV-1, respectively), their sequences contain only 36% pairwise and less than 10% overall sequence identity on average ([29] and protein domain family alignments database (PFAM) alignment of Herpes_UL35). No definitive function has been ascribed to HSV-1 VP26 or its equivalents, although it is believed to be involved in stabilizing hexons. Additionally, coexpressed VP26, VP5, and a scaffolding protein have been shown to assemble into complexes and translocate to the nucleus [30]. Mutants lacking VP26 grow normally in cell culture but are partially inhibited for growth in mice [31].

No high-resolution structure exists for VP26 or any of its equivalents nor are there any homologous structures. In fact, VP26 may exist in a monomer–dimer equilibrium, complicating high-resolution structural studies [29]. However, the combination of the X-ray and cryoEM structures did result in the localization of the density belonging to VP26 in hexon subunits [22,27]. As previously stated, VP26, a minor structural protein, only binds to VP5 in hexons; pentonal VP5s are instead bound, in the intact virion, to a large tegument protein, believed to be VP1–3. While structural analysis of the VP26 cryoEM density revealed no detectable secondary structure, circular dichroism studies indicated that bacterially expressed VP26, which had been solubilized with CHAPS and alterations to the ionic strength, was almost 80% β-sheet and only 13%–15% α-helical [29]. However, Desai et al*.*, using sequence-based secondary-structure prediction techniques, identified relatively little secondary structure, except for a helical region between residues 46 and 66 [32]. Therefore, VP26 may undergo dynamic structural changes between its bound and unbound states.

The difficulty in purifying native VP26 makes it difficult to determine its structure experimentally. As such, we have developed a hybrid modeling protocol using ab initio models generated with *Rosetta* in conjunction with cryoEM density assessment in an effort to improve the accuracy of the predicted structure of a VP26 core domain and to understand its interactions with other capsid proteins. In this paper, we present a VP26 core domain model, as well as an approach for determining/evaluating optimal structural models with the constraints imposed by a medium-resolution cryoEM density map. Such methodology may be generally applicable in modeling small proteins or domains from other macromolecular assemblies imaged by cryoEM.

## Results

### Hybrid Modeling with Cryo-EM

In this work, we have designed and implemented a protocol that augments the results from *Rosetta* ab initio modeling by using cryoEM density as a means for assessment. This methodology (described in detail in Materials and Methods) leverages the medium-resolution density envelope supplied by the cryoEM density map to provide the overall shape and density distribution in evaluating models when no structural homolog is known. In brief, a shape descriptor replaces the radius of gyration in a standard *Rosetta* modeling procedure, and a composite score based on twelve individual scores, including radius of gyration, is generated for every model. Once a set of decoys (possible models) has been generated, the individual models can be compared with a reduced representation of the cryoEM density map (a set of representative Cα atoms) using a two-way distance measure, and ranked. Initial representation and ranking of the models using a set of discrete points followed by a more exhaustive density-based search of the top models was used to minimize the computational time. From this, the most native-like model(s) can be selected and analyzed.

### Validation of a Known Structure, Hepatitis B Core Protein

**Figure 1:**
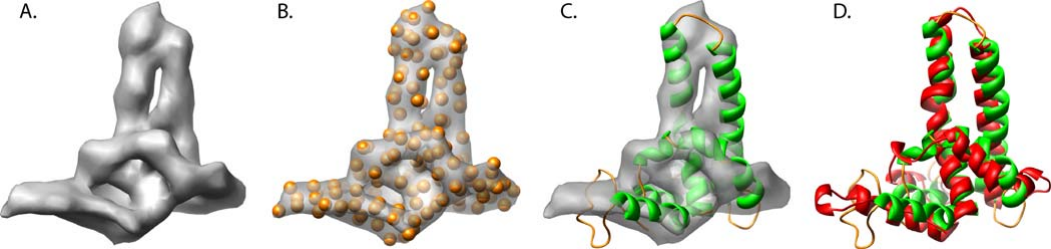
Modeling the Hepatitis B Capsid Protein The simulated density map of the hepatitis B virus capsid protein at 7.5-Å resolution is shown in grey (A). A reduced representation of the density map (orange spheres) is shown superimposed on the hepatitis B capsid protein density (B). The best decoy (6072) based on the described hybrid modeling protocol is shown superimposed on the simulated density (C). A comparison of the model structure (green-orange) and actual hepatitis B Virus capsid protein (red) is shown in (D).

In developing our hybrid modeling/evaluation protocol, the hepatitis B virus capsid protein, for which an X-ray crystal structure is available [33], was used as a test specimen. While hepatitis B was among the first subnanometer-resolution single-particle cryoEM reconstructions [34,35], the density maps are not available in the public cryoEM structure repository at the European Bioinformatics Institute. Therefore, a simulated density map of the hepatitis B capsid protein was generated from the crystal structure at 7.5-Å resolution with a sampling size of 2.3 Å/pixel (Figure 1A), similar to the authentic cryoEM map. Approximately 10,000 decoys were constructed and evaluated using the density map to re-rank the decoys scored with the standard *Rosetta* energy score (see Materials and Methods).

Individual models were ranked based on their similarity score to the reduced representation density map, where lower scores correspond to models that better agree with the reduced representation density and thus better agree with the native structure (see Materials and Methods). Model 6072 had the lowest two-way similarity score (3.25 Å) and correlated well (0.74) with the simulated density (Figure 1A–1C). The next best models, also based on the two-way similarity score, had larger values at about 3.4 Å. In analyzing the decoys without the aid of the simulated cryoEM density (based on the composite *Rosetta* score), model 4582 was identified as the top candidate model (Figure S1). However, when fit to the density, the correlation score of this model was significantly worse, 0.59, and had a two-way similarity score of greater than 5.0 Å.

Since the X-ray structure for the hepatitis B capsid protein is known (1QGT), additional model assessment could be accomplished through direct comparison of the X-ray structure to the individual models. Visual comparison of model 6072 reveals a striking structural similarity to the corresponding X-ray structure (Figure 1D), while model 4582 appeared to have little structural similarity. The RMS deviation between model 6072 and the X-ray structure was 6.22 Å for all 142 amino acids, while model 4582 had a RMS deviation of 9.15 Å when calculated with Matchmaker utility in University of California San Francisco's Chimera software [36]. Moreover, model 6072 had an RMS deviation of only 3.0 Å for the best 83 consecutive amino acids based on the *LGA* evaluation method [37]. Further comparison of all ~10,000 decoys with respect to the X-ray crystal structure in fact reveals that model 6072 generated by *Rosetta* was the most native-like model, as it had the lowest overall RMS deviation from the X-ray structure of any decoy. Based on this simulation, cryoEM density appears to be a valuable metric in the assessment of ab initio models.

*Dali* [38] and *DejaVu* [39], structure comparison tools, were used to further validate quantitatively the quality of the models. Individual models were used as probes in querying the Protein Data Bank (PDB) for the most similar structure, which included the hepatitis B structure. Both *Dali* (5.9 Å for 129 amino acids) and *DejaVu* (2.1 Å for 70 amino acids) returned 1QGT as the top-scoring structure for model 6072; however, model 4582 did not return 1QGT as a top structural homolog. As such, model 6072, selected using the cryoEM filtering method described (Materials and Methods), was indeed the most native-like model (i.e., the conformation of the subunit was the closest to the actual structure), confirming that this approach has the potential for selecting native-like structural models using the medium-resolution cryoEM density map.

### HSV-1 Capsid Protein, VP26

**Figure 2:**
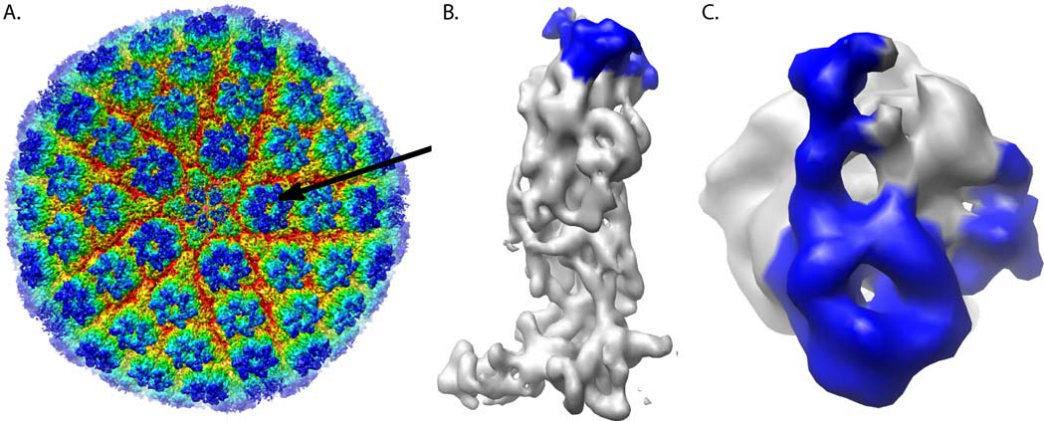
Structure of HSV-1 Hexon Subunit (A) The 8.5-Å resolution cryoEM structure of HSV-1 is shown radially colored. A hexon is indicated with an arrow. (B) The density of a single hexon subunit segmented from the 8.5-Å cryoEM map of the HSV-1 capsid is shown. (C) A top view of the hexon subunit is shown. VP26 shown in blue in (B) and (C) was isolated based on the difference map between the cryoEM density map of the hexon subunit and the VP5 upper-domain crystal structure.

As the resolution for the HSV-1 cryoEM map is relatively low (8.5 Å) [40], it is not possible to build a high-resolution structural model solely from the cryoEM density. Additionally, no structural homologues for VP26 are known. Thus, the aforementioned modeling protocol represents a potential, and perhaps the best, method for modeling VP26 in the context of the cryoEM density. A difference map between the X-ray structure of the upper domain of VP5 [27] and an averaged hexon subunit from the 8.5 Å cryoEM map of HSV-1 capsid (Figure 2A) has already established the region of density attributable to VP26 (Figure 2B and 2C). The main body of VP26 density rests on the top outer face of VP5 in the hexons. Two “arms” protrude from this main VP26 body and wrap around the sides of VP5. Analysis of a hexon subunit density map using *helixhunter* [6] and visual assessment revealed no definitive secondary structural elements in VP26 [26].

**Figure 3:**
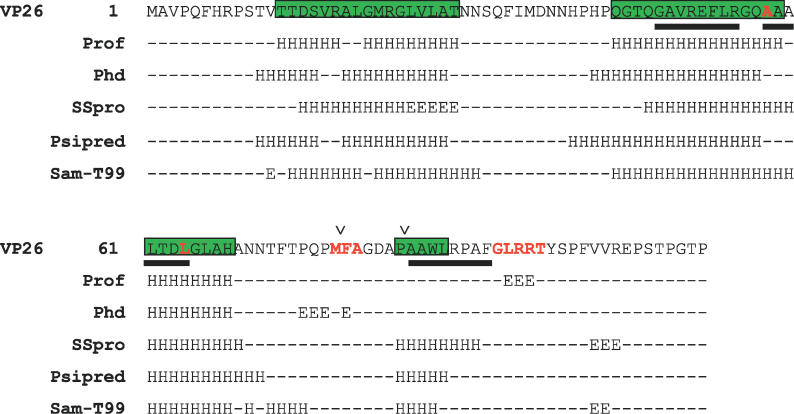
Secondary-Structure Prediction of VP26 Five secondary-structure prediction algorithms were used to compute the secondary structure (highlighted in green) of VP26. Mutations identified by Desai et al*.* [32] are highlighted in red. Helices based on model 8824 are represented by a black bar beneath the primary sequence. The locations on the VP26 sequence corresponding to the VP1–3 insertions (Figure 6) are labeled with “v” above the primary sequence.

Circular dichroism measurements previously estimated that a bacterially expressed and purified form of VP26 was predominantly β-sheet [29]. However, sequence analysis suggests that VP26 contains considerably less β-sheet content and possibly several small α-helices, while cryoEM density analysis revealed no α-helices longer than 2.5 turns or significantly large β-sheets [26,32]. Based on a consensus multiple secondary-structure prediction, VP26 appears to have two potentially α-helical regions (Figure 3). The first of these α-helical regions is at the N-terminus (residues 13–31) that likely contains two small α-helices. The second α-helical region is located in the middle of the VP26 sequence (residues 42–72) and contains either one large or two smaller α-helices. Although not as consistently predicted, the C-terminal region of VP26 may also contain a small α-helix.

Further analysis of the sequence and biochemical data also begins to provide evidence of the “gross” structure of VP26. An alignment of all VP26 homologues showed two fairly well-conserved regions (1–30 and 48–112) separated by a poorly conserved, gapped region between residues 31 and 47 of VP26. This organization is supported by previous mutagenesis that indicates residues 1–50 are not required for binding to VP5 or native-like function, while several mutations in the second half of VP26 affect VP26′s ability to bind to VP5 [32].

### Modeling a Subdomain of VP26

**Figure 4:**
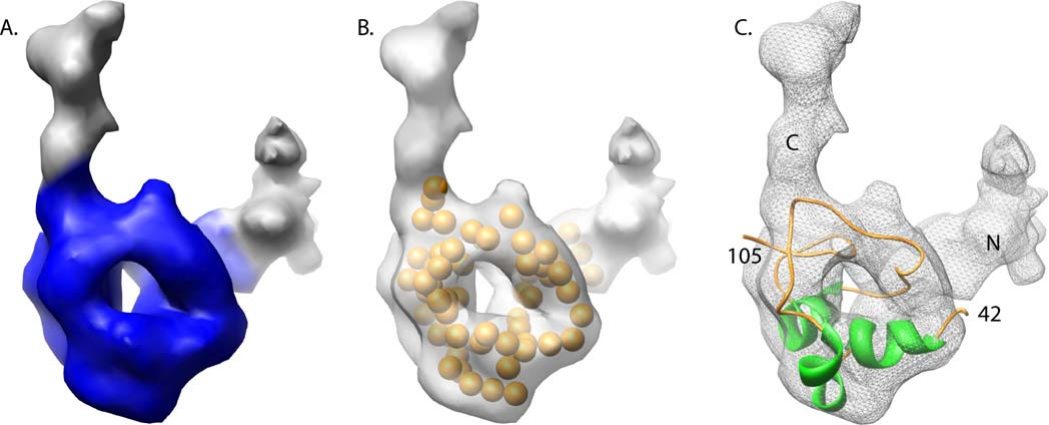
Modeling VP26 (A) The core domain of the segmented VP26 cryoEM density is colored in blue. (B) The pseudoatoms (orange spheres) assigned in the density-reduction step are shown superimposed on the cryoEM density of VP26. (C) The best model of the core domain (residues 42–105) generated by *Rosetta* and selected using cryoEM density constraints is shown fitted within the segmented VP26 density. The putative N-terminal and C-terminal density regions are labeled N and C, respectively.

Based on the aforementioned sequence, VP26 may contain two discrete structural and/or functional domains, the C-terminal one being responsible for interactions with VP5. Only this domain was used in constructing the model for VP26. Using the secondary-structure prediction as a guide, a slightly larger region, residues 42–112, was used in the modeling of this second (core) domain. The size of this domain corresponds to the approximate mass of the central VP26 density observed in the cryoEM map (Figure 4A and 4B). Approximately 20,000 decoys of the VP26 core domain were generated using the *Rosetta* protocol. These decoys were screened by both the cryoEM filtering and *Rosetta* scoring methods. The top decoys, based on both scores, were selected and fitted directly to the cryoEM density using *foldhunter* [6]. As suggested in the hepatitis B example, the cryoEM density is capable of discriminating a native-like model from a gallery of decoys. As such, selection of the final VP26 model was primarily based on the *foldhunter* cross-correlation score and the two-way similarity score, as well as being augmented by visual analysis and correlation with existing sequence/biochemical evidence.

As with the hepatitis B example, decoys selected based solely on the composite *Rosetta* energy score had worse two-way similarity scores than those screened against the cryoEM map. The top model (3554) based on the composite *Rosetta* energy score had a two-way similarity score of 5.16 Å, close to the average score (5.19 Å) for the gallery of decoys.

However, unlike the hepatitis B, there was no singularly best structure based on the two-way similarity score with the reduced cryoEM representation (Figure S2 and Table 1). Rather, 25 decoys had a two-way similarity score better than 4 Å. All these decoys appeared to share a similar overall composition, based on visual inspection, although the models were sufficiently different in their structure. Of note, these 25 models, when aligned to each other using Chimera's Matchmaker, had RMS deviations similar to the resolution of the experimental cryoEM density map (~8.0 ± 1.5 Å). Of these 25 decoys, the top ten appeared to be relatively self-consistent, having RMS deviations between 3.77 Å and 3.92 Å amongst each other (Figure S3). Additionally, when exhaustively fit to the density with *foldhunter*, the correlation scores of these top ten models were again very similar, ranging from 0.58 to 0.65, respectively (Table 1). The remaining 15 models had lower correlation scores and larger RMS deviations amongst each other.

**Table 1:**
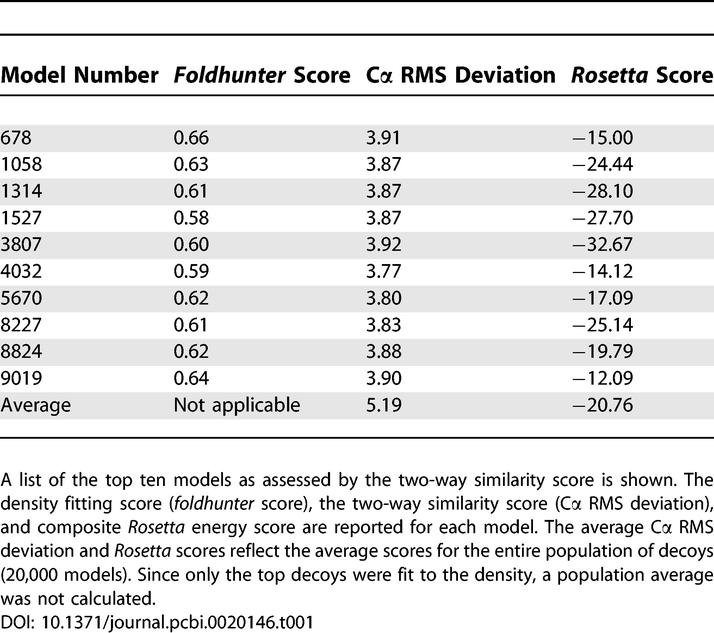
VP26 Decoy Statistics

To further differentiate among the possible models, the decoys were analyzed in the context of the VP26 density by itself and in association with the hexon subunit density from the original cryoEM map. Of the top models, seven models had the termini pointing out of the VP26 density, making them less likely core domain–model candidates. Model 8824 had a highly ranked two-way similarity score and fitting score (fifth and fourth best, respectively), as well as having “good” orientations for the two VP26 domain termini. As such, model 8824 (Figure 4C), with a similarity score of 3.88 Å and a correlation score of 0.62, was chosen as the most probable native-like structure for the core domain of VP26. Again, it is worth noting that the structural variation of the top 25 models, assessed by calculating the RMS deviation with respect to model 8824, were on a par with the resolution of the experimental cryoEM density map.

As with the hepatitis B example, the VP26 core domain model 8824 was used to search against the PDB for a homologous structure. No homologous structure was identified with *DejaVu* or *Dali*. As such, the modeled core domain of VP26 represents a novel fold that contains three short α-helices, as predicted by secondary-structure prediction (Figures 3 and 4). These α-helices are small enough that they would not be identified through analysis of the 8.5 Å cryoEM capsid density map, which requires a minimum α-helix length of ~2.5 turns [6]. In model 8824, the N- and C-termini of the VP26 core domain (residues 42 and 105, respectively) extend towards the two VP26 “arms.” It was not possible to model the C-terminal seven amino acids (106–112) by *Rosetta,* as they appeared to be disordered. However, these residues can easily be accommodated by the remaining cryoEM density along the extended arm. The N-terminal residues (1–41), which were not modeled, likely extend down the other arm. Density in this region, which extends below the core domain of VP26, could account for a large number of these residues. As the two termini appear to wrap around VP5, it is likely that interactions between neighboring VP26 molecules occur exclusively at the N- and C-termini of VP26.

### Interactions with VP5

**Figure 5:**
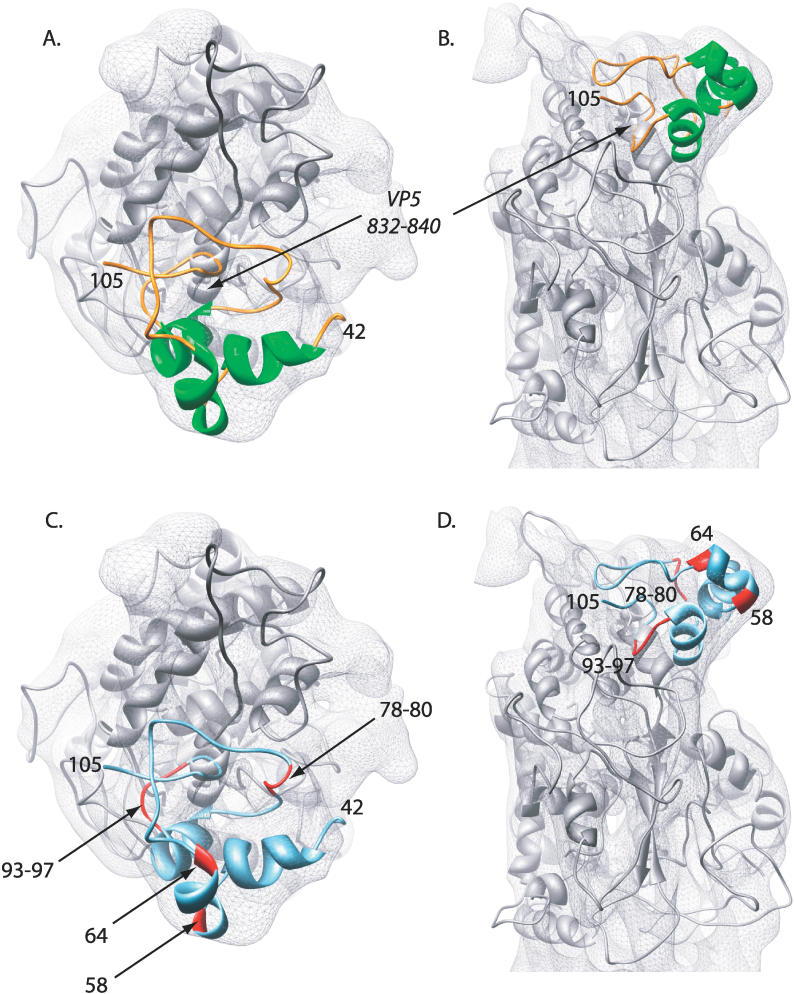
VP26 Interactions (A,B) The VP26 core domain model is shown in the context of the VP5 upper-domain X-ray structure (grey) in a top view and a side view, respectively. The α-helix in VP5 (832–840) that interacts with VP26 is labeled. (C,D) Mutations (labeled with arrows and highlighted in red on the ribbon diagram) in VP26 (cyan blue) that affect binding to VP5 as described by Desai et al. [32] are shown in top and side views, respectively.

By fitting the VP26 core domain model and the X-ray structure of the upper domain of VP5 to an HSV-1 hexon subunit map derived from the cryoEM density, it is possible to observe potential interactions between the two proteins (Figure 5A and 5B). The interface between the two proteins is seen to be dominated by loops, except for one α-helix on VP5 (832–840, FDRVYATLQ).

Aside from a small clash between a loop in VP26 and an α-helix in VP5, the surfaces of the proteins appear to have a relatively complementary interface (Figure S4). Both the VP26 core domain and the upper domain of VP5 are dominated by hydrophobic residues at their interface. However, two small patches of charged residues are located near each of the VP26 arms (Figure S3). In the first of these VP26 patches, near the N-terminus of the model, residues R51, E52, R55, and D82 are proximal to a patch of charged residues on VP5 (R834, D953). The second charged patch on VP26 (R89, R95, R96, R104, E105), near the C-terminus, also appears to be proximal to another charged region in VP5 (D775, D833, H899, E903).

Site-directed mutagenesis has previously identified functionally important residues in VP26 [32]. In particular, mutations at M78, F79, A80, G93, L94, R95, R96, and T97 eliminated binding of VP26 to VP5 in capsids and mutants in A58, and L64 reduced binding of VP26 to VP5 in capsids (Figure 3). Additionally, mutations at F79 and G93 altered the efficiency of translocation to the nucleus, suggesting that initial interaction of VP26 and VP5 may occur in the cytoplasm through F79 and G93. It should also be noted that these residues are the most conserved in all of the members of the *Herpesviridae*. Residues 78–80 are absolutely conserved while residues 93–97 have only one identified substitution, lysine replacing arginine at position 96 (from PFAM Herpes_UL35).

Significantly, the regions containing mutations that resulted in elimination of VP26 binding, residues 78–80 and 93–97, lie at the proposed interface between VP26 and VP5 (Figure 5C and 5D). Residues 78–80 (M78, F79, A80) are adjacent to two VP5 loops (N341, M342, A872, N873, T874), while residues 93–97 of VP26 appear close to another VP5 loop (V771, A772, T773). The two mutations at A58 and L64, which resulted only in decreased binding of VP26 to capsids, were on an α-helix although not at the VP26/VP5 interface.

### Structural Homology: VP26 and the Large Tegument Protein

**Figure 6:**
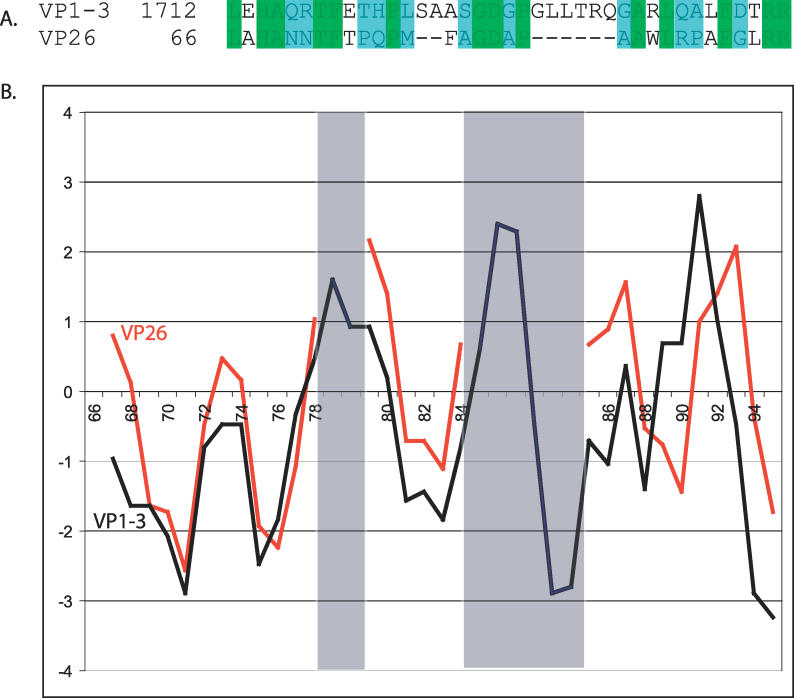
Common Sequence Motif in VP26 and VP1–3 A sequence alignment of VP26 and VP1–3 is shown in (A). Identical residues are shown in green, while conserved residues are shown in cyan blue. In (B), the hydropathy profiles of this region from VP26 (red) and VP1–3 (black) are plotted and labeled with respect to the VP26 sequence. The shaded panels indicate the regions of insertion found in the sequence alignment.

As previously mentioned, VP26 binds to VP5 in hexon subunits but not in penton subunits. However, it has been shown that a tegument protein, most likely VP1–3, associates with the penton VP5 subunit at approximately the same interface that VP26 interacts with hexon VP5 [41]. It has also been speculated that the differential affinity of VP26 and the tegument protein for hexon and penton subunits, respectively, is likely based on steric and conformational considerations [27,42]. Locally aligning the primary sequence of VP26 and VP1–3 revealed a region of similarity (Figure 6A). Residues 66–96 of VP26, which make up nearly the entire interaction surface with VP5, share 45.2% identity (80.6% similarity) to residues 1712–1751 of VP1–3. Additionally, the alignment of all VP26s and a PSI-Blast alignment of VP1–3s show this region is highly conserved in both proteins. Based on this alignment, there appears to be two differences between the VP26 and VP1–3 sequences: a two-residue insertion between residues 78 and 79 of VP26 and a larger six-residue insertion between residues 84 and 85 of VP26 (Figures 3 and 6). Hydrophobicity plots of these regions in VP26 and VP1–3 show almost identical character, differing only in the gapped regions (Figure 6B). In the context of the VP26 core domain model, these gaps are located along a portion of the probable VP26–VP5 interface. Based on this, it is reasonable to speculate that the local structure for these regions in VP26 and VP1–3 are similar and interact with VP5 using the same mechanism.

## Discussion

Large macromolecular machines are underrepresented in the PDB, as their size and complexity can make it difficult to study them structurally [43,44]. Therefore, proteins from these macromolecular complexes make a relatively small contribution to our knowledge of fold space. Because of this, construction of homology models for macromolecular components may be based on suboptimal templates or may not be possible at all. Since ab initio modeling does not require structural templates, it offers great promise in modeling components from large macromolecular assemblies. Though it is potentially feasible to generate models for the components/domains of macromolecular assemblies, a lack of macromolecular assembly structures may bias the structural models towards those of single, soluble proteins, potentially ignoring the extensive interactions within a complex. Nevertheless, the major challenge in ab initio modeling still lies in the identification of the most probable native-like models from a large number of potential models.

Currently, there are many methods for assessing models from a gallery of decoys, with varying degrees of success. However, most of the metrics to assess the individual decoys are based on single, soluble, globular proteins. The assessment method used in the work presented here evaluates the models in the context of a biologically active entity. As such, the selected model is reflective of the protein in a biologically active conformation, which may be different from a biochemically isolated state. Our approach is to include the maximum amount of biological and structural information in selecting the best decoys in a biological context.

While limited to medium resolutions, cryoEM can capture the overall structure of a complex and its components in well-defined chemical or biological states. In this work, we have utilized this “low-resolution” information to select for the most probable native-like models from the ensemble of structures generated during ab initio modeling. By using a reduced representation density map, we were able to rapidly evaluate a large number of potential models for further analysis. In the case of VP26, exhaustive fitting of all 20,000 models to the original cryoEM density map using standard density matching methods, such as *foldhunter* [6], would have taken more than 1,500 cpu hours, while the calculation of the aforementioned similarity score for the same number of decoys required less than one hour on a single cpu. Coupled with the exhaustive correlation-based search of the top models, the entire method required less than three cpu hours. In addition to allowing for the rapid evaluation, the advantages of this approach were illustrated with the hepatitis B example, where it was possible to identify the most native-like model from a set of ~10,000 decoys, something the composite *Rosetta* energy score was unable to do.

This work only represents the initial application of cryoEM to ab initio modeling. It is likely that this and similar methods will become increasingly valuable as the number of cryoEM structures and their complexity continue to grow [44,45]. Similar approaches have already been used with comparative modeling, in which cryoEM density was shown to capture low-resolution structural features and facilitate model selection [14,15]. As such, similar techniques may become commonplace in the near future for the evaluation of structures in macromolecular complexes.

### Modeling Limitations and Accuracy

While seemingly producing credible models for VP26 and the hepatitis B capsid protein, the described hybrid modeling/assessment method is not without its limitations. At present, ab initio modeling is generally limited to proteins or domains of fewer than 300 amino acids. Unfortunately, the use of cryoEM is only involved in the assessment of decoys and cannot remove this limitation. Therefore, this type of hybrid approach is only appropriate for modeling individual proteins or domains of this size that can be clearly delineated from the cryoEM density maps of the macromolecular complex. This approach is therefore subject to the limitations imposed by the intrinsic properties of the cryoEM density map and the ability to define subunit boundaries within it. If the resolution and/or quality are insufficient to accurately identify the boundaries of the individual components, domains, or structural features in macromolecular complexes, a cryoEM density map will be incapable of accurately discriminating amongst different decoys. The true power of the cryoEM density selection is in the intrinsic shape and density distribution of the protein in question.

As indicated, the resolution and accuracy of the cryoEM density map is paramount for accurate model selection. In the hepatitis B example, simulated cryoEM density was used to select the top model among the gallery of decoys, which was also the most native-like decoy, when compared with the X-ray structure, generated by *Rosetta*. This, of course, is the ideal situation and will not always be the case. Generally though, more accurate models are more likely to be selected by higher-resolution cryoEM density maps. Additional high-resolution features, such as secondary structure, may also be used in model selection or may even be incorporated into the decoy generation phase in *Rosetta*.

For VP26 from the HSV-1 capsid cryoEM structure, its resolution (8.5 Å) essentially limits the accuracy at which the most native-like model may be selected. The top models, when aligned to the VP26 core domain density map, possess RMS deviations close to the resolution of the cryoEM density map. Thus, the overall ability to identify the most native-like decoy in VP26 is directly related to the resolution and quality of the cryoEM density map, and as such the interpretation of the model should be judged accordingly. By using other biochemical and genetic information, the combined bioinformatics would increase the resolving power of this approach as demonstrated in this study.

While the cryoEM density information should provide a metric for selecting a protein or domain, it may not be any more useful than standard shape descriptors such as radius of gyration for small, globular folds. In the case of compact globular folds, the cryoEM density may not be descriptive enough to select the best possible model for the decoys. With regard to the two structures modeled in this work, the core domain model of VP26 was considerably more globular and compact than the hepatitis B capsid structure. This may account for the different score distributions in these two cases and the difficulty in selecting the VP26 model based solely on the two-way similarity score. Furthermore, different secondary-structure composition, specifically β-sheets which are only visualized in relatively high-resolution cryoEM density maps, could also alter the effectiveness of cryoEM density in assessment of models. Therefore, as with any modeling technique, scoring metrics should be viewed as guides in selecting the best model, but visual interrogation of the models and correlation with biochemical data is necessarily the best judge of model accuracy.

Not to be overlooked in evaluating models in the context of the density is the ability to accurately localize coordinate data to the density map. Traditionally, fitting techniques, such as *foldhunter* [6], are based on exhaustive correlation-based techniques, which are extremely sensitive but fairly slow. In this work, we used a reduced representation of the cryoEM density map, a set of pseudo-Cα atoms, to improve the computational efficiency of decoy evaluation. While this type of representation allows for a rapid screening of decoys, it does not replace the sensitivity of an exhaustive correlation-based fitting technique. This evaluation merely provides an initial ranking to reduce the number of potential decoys for further evaluation. Differences in different data-reduction algorithms (K-means, vector quantization, etc.) will thus not likely affect the overall outcome of model evaluation. In this work, we observed comparable placement of pseudoatoms irrespective of the method used in discritization of the hepatitis B density map (Figure S1). While the two-way score varied slightly with the discritization method, the difference was negligible and did not affect the relative rankings of the individual models.

### VP26 Structure

At present, no structure for VP26, its herpesvirus analogues, or a structural homolog is known. In fact, previous biochemical work has suggested that HSV-1 VP26 is not very soluble and exists in a monomer–dimer equilibrium [29]. Structural studies have alternatively hypothesized that VP26 forms hexamers during viral assembly, which could account for VP26′s preference for hexons [22]. It has also been reported that VP26 must be associated with VP5 to enter the nucleus, where viral capsid assembly occurs [30]. As such, soluble, unbound VP26 may in fact be in a different conformation than VP5-associated VP26. Differences between circular dichroism measurements of CHAPS solubilized, bacterially expressed VP26, and the sequence and structural observations may indicate such conformational variability. Thus, our hybrid modeling approach, which leverages cryoEM density, may represent the best and at present possibly the only feasible way to determine a structural model of HSV-1 VP26 bound to the virus capsid.

In the modeling of VP26, the first 41 amino acids were excluded, as this likely represents a discrete domain. This portion of the protein had previously been shown to be nonessential for the interaction with VP5 [32]. Furthermore, the cryoEM density of VP26 appears to indicate the presence of a globular domain with one small and one large arm extending away from the main density. The model of the VP26 core domain appears to fit well into the main density, with the two termini pointing towards the individual arms. The arm, which was assigned to the N-terminus, is relatively large and can probably be considered as an independent domain. While this N-terminal density is substantial and can accommodate most of the first 41 amino acids, it is possible that portions of N-terminus may extend towards the neighboring VP26 subunit, and our segmentation of a single VP26 subunit may not be entirely accurate due to the cryoEM map resolution. This region of density therefore may not fully reflect the entire N-terminus of VP26; however, its implication in modeling the VP26 core domain is negligible.

Since no other structural data is available for VP26 or related structures, mutational and structural information can be used to validate the model. It appears that the core domain model of VP26 is consistent with the mutational data, since mutants that abolish VP26′s ability to bind to VP5 are placed at the interface of these two proteins. However, two mutations (amino acids 58 and 64) that reduce the affinity of VP26 for VP5 are found in one of the α-helices that form the outer surface of VP26. Since these mutations are in an α-helix, it is possible that mutations at these residues affect the affinity of VP26 for VP5 by altering the overall VP26 structure.

### Molecular Interactions

Structurally, the VP26 core domain model appears to fit well within the cryoEM density while allowing for the accommodation of the remaining unmodeled residues. Moreover, the predicted interacting surface of VP26 appears consistent with what is known about the VP5 interface region. While the model itself was selected using the cryoEM density, VP26 was modeled as an independent structural unit. As such, no bias towards the VP5 interface was enforced. The fact that the predominantly hydrophobic regions and charged regions of VP26 and VP5 appear to be complementary further enforces the plausibility of the model. This is further augmented by the relative agreement of the mutational data for VP26. The fitting to the cryoEM map produced only one slight clash between the VP26 core domain model and the VP5 upper-domain structure. This clash could reflect the potential for structural variation when VP26 and VP5 interact or could simply be an artifact due to the relatively low resolution of the cryoEM density map. Furthermore, this region of VP26 is in a loop, for which model accuracy is likely to be significantly worse than in regions of α-helices or β-sheets. Regardless, this small clash is likely to be relatively insignificant in terms of the overall structure and function of VP26.

While binding of VP26 to VP5 is specific to hexons, the equivalent VP5 interface in pentons is occupied by a tegument protein (thought to be VP1–3) [22,29,41]. The different binding specificities probably reflect the varying structures and spatial arrangements of the VP5 subunits in hexons and pentons, respectively [26,27]. However, of interest in the context of the work described here is the similarity in the footprints occupied by VP26 and VP1–3 on the VP5 subunits. Sequence analysis reveals that the essential binding region in VP26 shares a high degree of similarity to a short sequence in VP1–3. The high sequence relatedness and similar hydrophobicity profiles suggest that the local structure of this portion of VP1–3 probably resembles that of VP26. If this region of VP1–3 is involved in binding to VP5, it might be expected to create an equivalent interface to that seen with VP26. Local differences due to imperfect conservation and the influence of steric considerations and oligomeric states of VP5 could then account for the differential binding to hexons and pentons through a similar interaction interface.

### Conclusion

While the results described here represent only a first attempt to model the core domain of VP26, they nevertheless provide useful insights into the probable organization of VP26 and its interactions with VP5. Importantly, this model provides the first evidence to our knowledge of VP26 structure from which biological and additional structural experiments could be carried out to further confirm the model and the proposed mechanism for interaction. Moreover, the results presented here, in combination with similar work in comparative modeling [14,15], illustrate the potential efficacy and credibility of hybrid modeling approaches. Of particular note is that by using cryoEM density, along with biochemical and sequence data, it is possible to produce models in the context of the biologically relevant macromolecular assemblies. With the ever-growing availability of cryoEM density maps, hybrid methods such as this will likely become increasingly important tools in deciphering macromolecular structure and function.

## Materials and Methods

### Density representation.

To efficiently integrate cryoEM density into the modeling protocol, we adopted a reduced representation based on the density map to faithfully characterize the density distribution. This representation allows for the rapid comparison of a large number of models that will be generated during the model-building process. A similar reduced-density representation strategy has been previously used in the fitting of coordinate data to cryoEM density maps [10] and normal mode computations in cryoEM density maps [46]. Essentially, a defined number of points (pseudoatoms) are assigned within the density map to faithfully approximate the density distribution. Assignment of these points can be done using a variety of algorithms including vector quantization, K-means clustering, or direct, threshold-based techniques. For the purposes of this work, pseudoatom assignment was done using K-means clustering in EMAN [47], although other methods from Situs [10] and EMAN produced similar results in representing a cryoEM density map accurately at a given density threshold. In each of these approaches, the pseudoatom corresponds to a unique density segment, which may not represent a single, discrete amino acid at the current resolutions obtained by cryoEM. Only the number of amino acids, corresponding to the number of pseudoatoms and a density threshold that approximates the mass of the protein in question, was used in the generation of the pseudoatoms from *segment3d* in EMAN [47].

The reduced-density representation corresponds to the density map “constraints” during the alignment and evaluation of the predicted decoys (alternative models). In this application, the points from the reduced representation are conceptually regarded as pseudo-Cα atoms but without identity, i.e., the amino acid type and position in the sequence. Figures 1B and 4B depict the pseudoatom representations in the hepatitis B capsid protein and the VP26 core domain, respectively.

### Density-based model assessment in ab initio structure prediction.

In typical *Rosetta* predictions, large numbers of decoys are generated and ranked based on a variety of scoring functions, with varying degrees of success [48]. Here, the cryoEM density map is used as a new type of scoring function, based on the overall density shape. The standard *Rosetta* composite score consists of 12 independent scores, including the radius of gyration which preferentially selects more compact models. In this work, we attempt to improve the score represented by radius of gyration with a more targeted shape-matching score by comparing the models with the medium-resolution cryoEM density map. To rank decoys using the cryoEM density map, a three-step process was performed:

*Rigid body alignment of decoys to the density map*. Ellipsoid fitting [6] was used to align decoys to the reduced-density representation. The rotation matrix between the principal axes of the ellipsoids was calculated using a closed-form formula. The decoy coordinates were transformed to match the density map.

*Calculating a similarity score.* A score was computed based on the sum of the distances of every Cα atom to the closest point in the reduced-representation density map after alignment (step number 1). Additionally, the distances from every point in the reduced representation density map to its closest Cα atom in the decoy was calculated and summed. This two-way distance measure, the average of the two distance measures, is used to assess map/decoy agreement and prevent abnormally compact decoys from incorrectly scoring well.

*Rank*. The scores of all the decoys from step number 2 were ranked. The top decoys, which have the smallest similarity scores, were chosen as the most favorable models for further investigation. Further density-based fitting of the top models to the original cryoEM density was performed using *foldhunter*.

### Validation with hepatitis B.

A single subunit of hepatitis B capsid protein (142 amino acids) was blurred to produce a 7.5-Å resolution density map using the *pdb2mrc* program in EMAN [47]. The density map was then quantized using 142 pseudoatoms using k-means clustering in the EMAN program *segment3d*.

The sequence of this protein was subjected to *Rosetta* prediction, for which 10,000 backbone-only decoys were generated. Decoys were scored using the above-described two-way density-similarity metric. Comparison of the best model and the native structure was done using the *LGA* evaluation method [37], *Dali* [38], and *DejaVu* [39].

### VP26 density segmentation.

VP26 was segmented by fitting the VP5 upper-domain structure to a single hexon subunit from the 8.5-Å resolution HSV-1 structure as described previously** [27]. Essentially, a mask can be created from the blurred VP5 density and applied to the hexon subunit. The remaining density at the outermost portion of the hexon subunit was designated as VP26.

### Sequence analysis.

The sequences for VP26 and VP1–3 were obtained from Swissprot. The sequence alignment of known VP26 homologues in *Herpesviridae* was obtained from PFAM [49], while the sequence alignment of VP1–3 was generated using Psi-Blast [50]. Local alignment of VP26 with VP1–3 was done using *LALIGN* [51]. Hydrophobicity plots were generated based on the Kyte and Doolittle option with a three amino acid–window size in the *ProtScale* program available from http://www.expasy.ch. Secondary-structure prediction was done online using *JPRED* [52], *Psipred* [53], *Predator* [54], *PhD* [55], and *SSPRO* [56].

### Modeling a subdomain of VP26.

Based on the aforementioned sequence analysis and mutational data [32], the VP26 primary sequence was divided into two domains; residues 1–41 and 42–112. The second, larger domain (core) was assumed to comprise the relatively globular domain of the VP26 density. Seventy pseudoatoms, corresponding to the number of amino acids to be modeled, were generated using the *segment3d* program in EMAN.

Twenty thousand decoys were generated for the VP26 core domain (residues 42–112) using the *Rosetta* prediction method. All models were then scored using both the standard composite *Rosetta* energy score and the two-way similarity score. Of these models, the top models based on the *Rosetta* score and the two-way similarity score metric, which had better than a 4-Å similarity score with the VP26 pseudoatoms, were fit to the VP26 cryoEM density using *foldhunter* [6]. The *foldhunter* score, similarity score, *Rosetta* score clustering, and visual analysis were then used concurrently to identify the best model.

## Supporting Information

Figure S1 Modeling Hepatitis B The density map for the simulated hepatitis B capsid protein is shown superimposed on the pseudoatoms (orange), calculated using (A) vector quantization and (B) K-means. The X-ray structure of the hepatitis B virus capsid protein is shown as a series of Cα atoms (C), rainbow-colored blue (N-terminus) through red (C-terminus), and as a full-ribbon model (D). The top model selected using cryoEM density is shown in (E), while the model with the best *Rosetta* score is shown in (F).
(4.6 MB PDF)

Figure S2 Plot of the Two-Way Similarity Score for the VP26 Core Domain Decoys The scores for all decoys having a two-way similarity score less than 8 Å RMSD with the reduced representation density have been plotted. The average of these ~12,000 decoys was 5.19 Å (green line). A zoomed in view (red box) of the top 250 decoy scores is shown in the subplot.
(1.3 MB PDF)

Figure S3 VP26 Core Domain Decoys A gallery of the top ten decoys using the two-way similarity score is shown. Additionally, model 3554, which has the best *Rosetta* energy score, is shown in the upper right corner of the gallery. The best model (8824) is superimposed on the pseudoatoms constructed from the VP26 density map. All models are shown as fit to the density using *foldhunter* and viewed as in Figure 4.
(6.5 MB PDF)

Figure S4 Capsid Protein Interactions The interactions of VP26 and VP5 are shown in relation to electrostatic potential (row 1) and hydrophobicity (row 2). Positive residues are colored in blue, negative residues are colored in red, and hydrophobic residues are shown in salmon.
(5.2 MB PDF)

### Accession Numbers

For materials mentioned in this paper, the accession numbers from the Protein Data Bank (PDB) (http://www.rcsb.org/pdb) are: for hepatitis B virus capsid protein (X-ray crystal structure) (1QGT); and for VP5 upper-domain crystal structure (1NO7). The accession number from PFAM (http://pfam.wustl.edu/) is for VP26 homologues (Herpes_UL35). Accession numbers from Swiss Prot (http://swissprot.org) are: VP26 (P10219) and VP1–3 (P10220).

## Abbreviations

cryoEM: electron cryomicroscopy
HSV-1: herpes simplex virus type 1
PDB: Protein Data Bank
PFAM: protein domain family alignments database
