# Supplementary figures and images for: Ab Initio Modeling of the Herpesvirus VP26 Core Domain Assessed by CryoEM Density
Source: PLoS Comput Biol. 2006 Oct 27;2(10):e146. doi: 10.1371/journal.pcbi.0020146 (PMC1626159; doi:10.1371/journal.pcbi.0020146)

A.

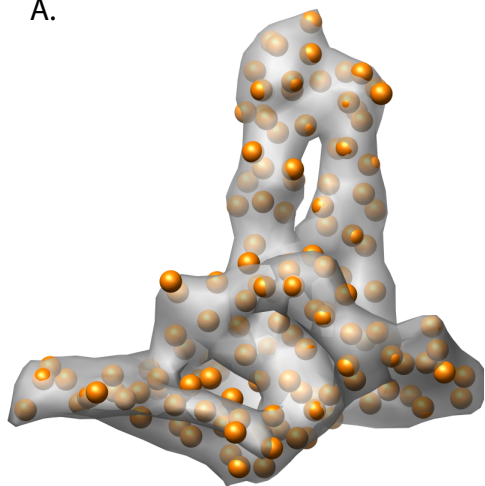

B.

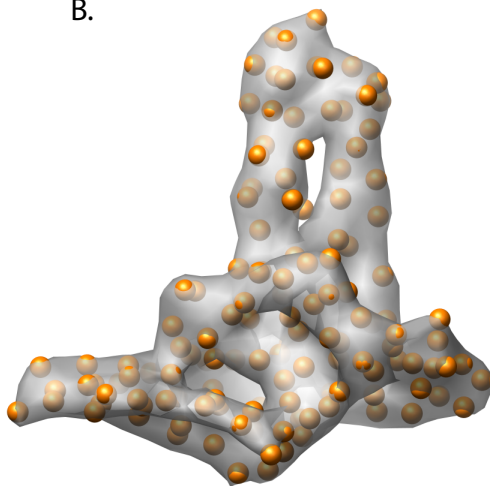

C.

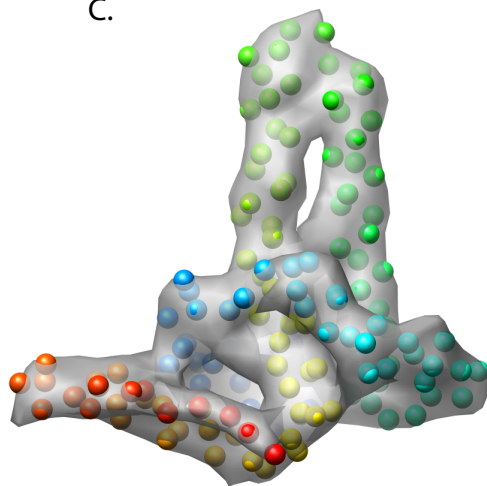

D.

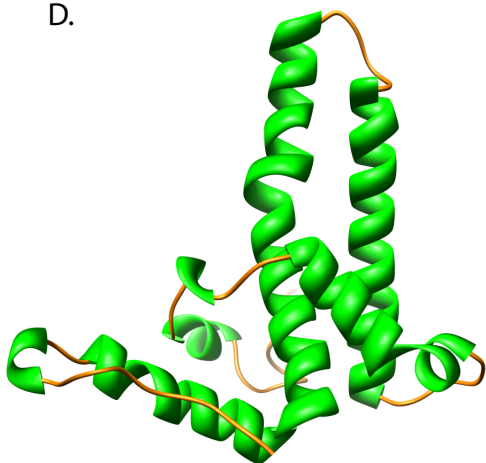

E.

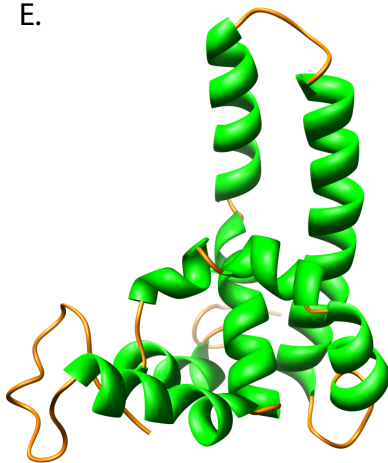

F.

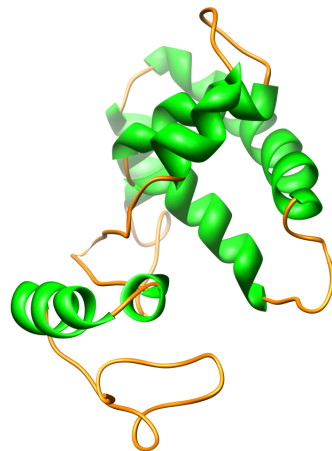

Supplement: Figure S1 — The density map for the simulated hepatitis B capsid protein is shown superimposed on the pseudoatoms (orange), calculated using (A) vector quantization and (B) K-means. The X-ray structure of the hepatitis B virus capsid protein is shown as a series of Cα atoms (C), rainbow-colored blue (N-terminus) through red (C-terminus), and as a full-ribbon model (D). The top model selected using cryoEM density is shown in (E), while the model with the best Rosetta score is shown in (F). (4.6 MB PDF) [file pcbi.0020146.sg001.pdf]

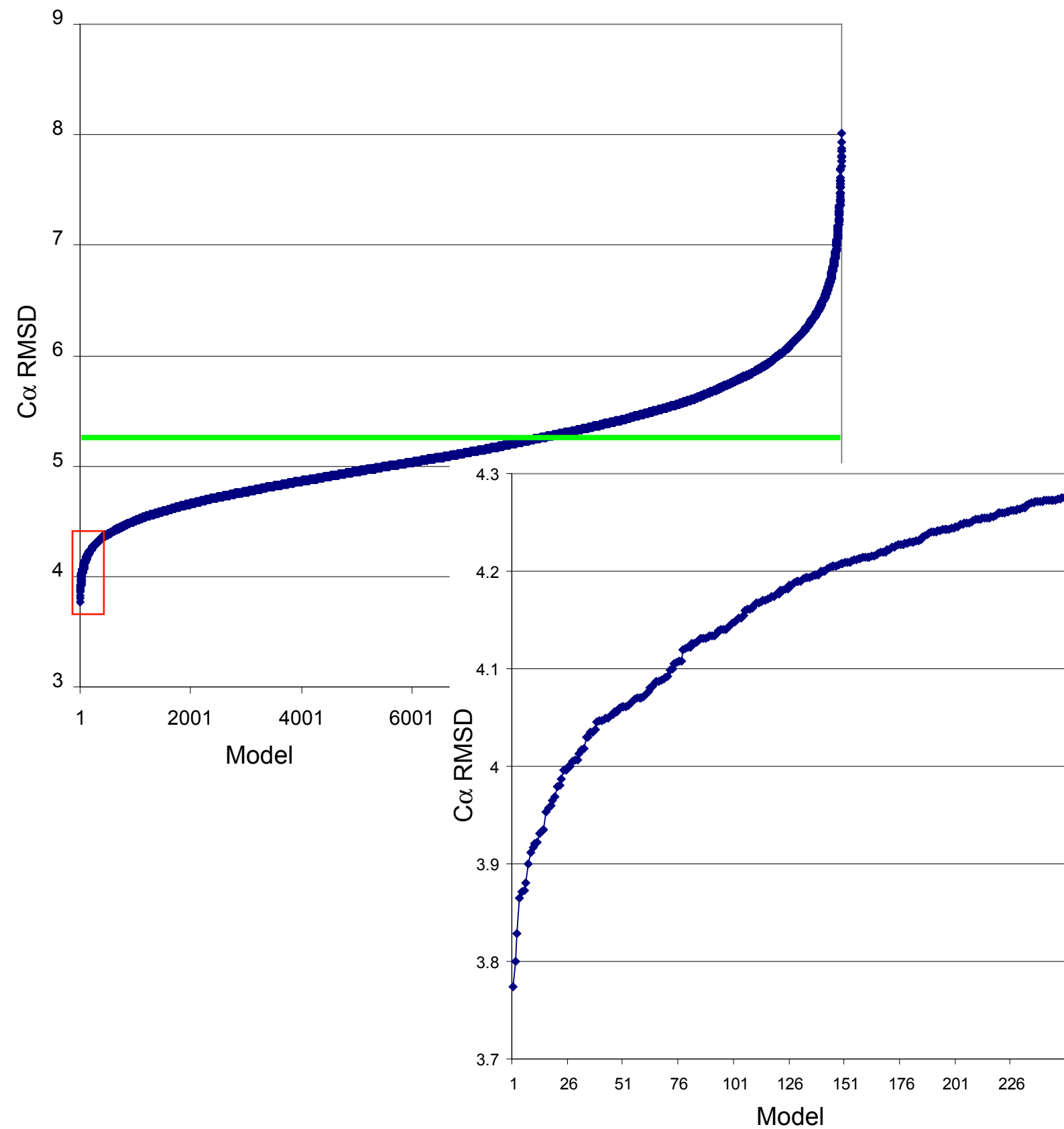

Supplement: Figure S2 — The scores for all decoys having a two-way similarity score less than 8 Å RMSD with the reduced representation density have been plotted. The average of these ~12,000 decoys was 5.19 Å (green line). A zoomed in view (red box) of the top 250 decoy scores is shown in the subplot. (1.3 MB PDF) [file pcbi.0020146.sg002.pdf]

model 8824

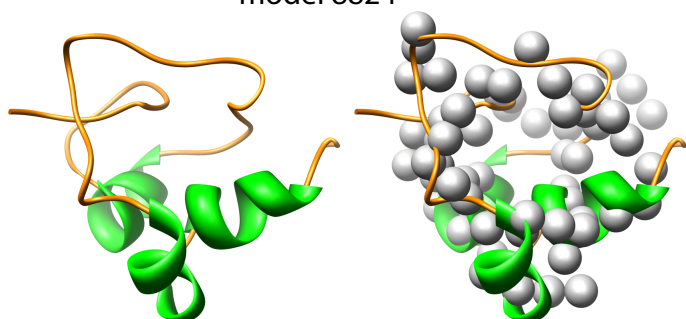

model 3554

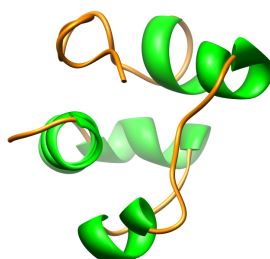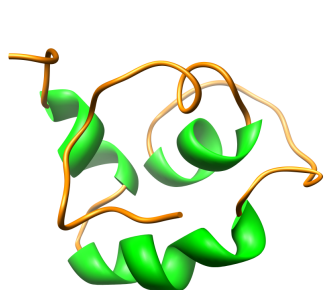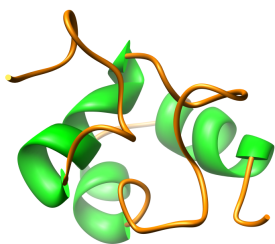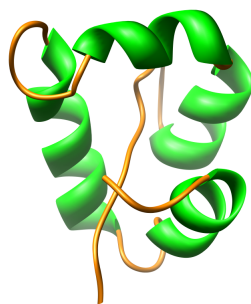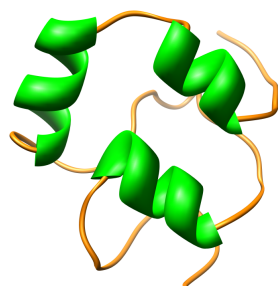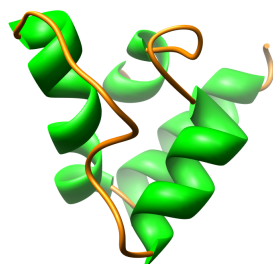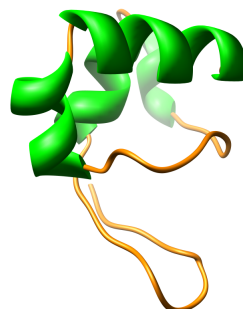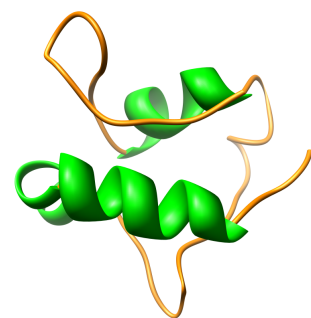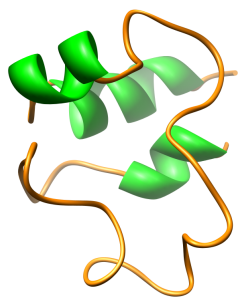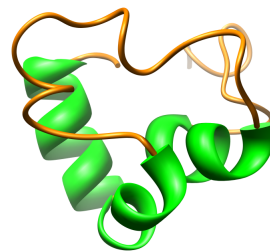

Supplement: Figure S3 — A gallery of the top ten decoys using the two-way similarity score is shown. Additionally, model 3554, which has the best Rosetta energy score, is shown in the upper right corner of the gallery. The best model (8824) is superimposed on the pseudoatoms constructed from the VP26 density map. All models are shown as fit to the density using foldhunter and viewed as in Figure 4. (6.5 MB PDF) [file pcbi.0020146.sg003.pdf]
