# Supplementary material for: Ab Initio Modeling of the Herpesvirus VP26 Core Domain Assessed by CryoEM Density
Source: PLoS Comput Biol. 2006 Oct 27;2(10):e146. doi: 10.1371/journal.pcbi.0020146 (PMC1626159; doi:10.1371/journal.pcbi.0020146)
Supplement: Figure S4 — The interactions of VP26 and VP5 are shown in relation to electrostatic potential (row 1) and hydrophobicity (row 2). Positive residues are colored in blue, negative residues are colored in red, and hydrophobic residues are shown in salmon. (5.2 MB PDF) [file pcbi.0020146.sg004.pdf]

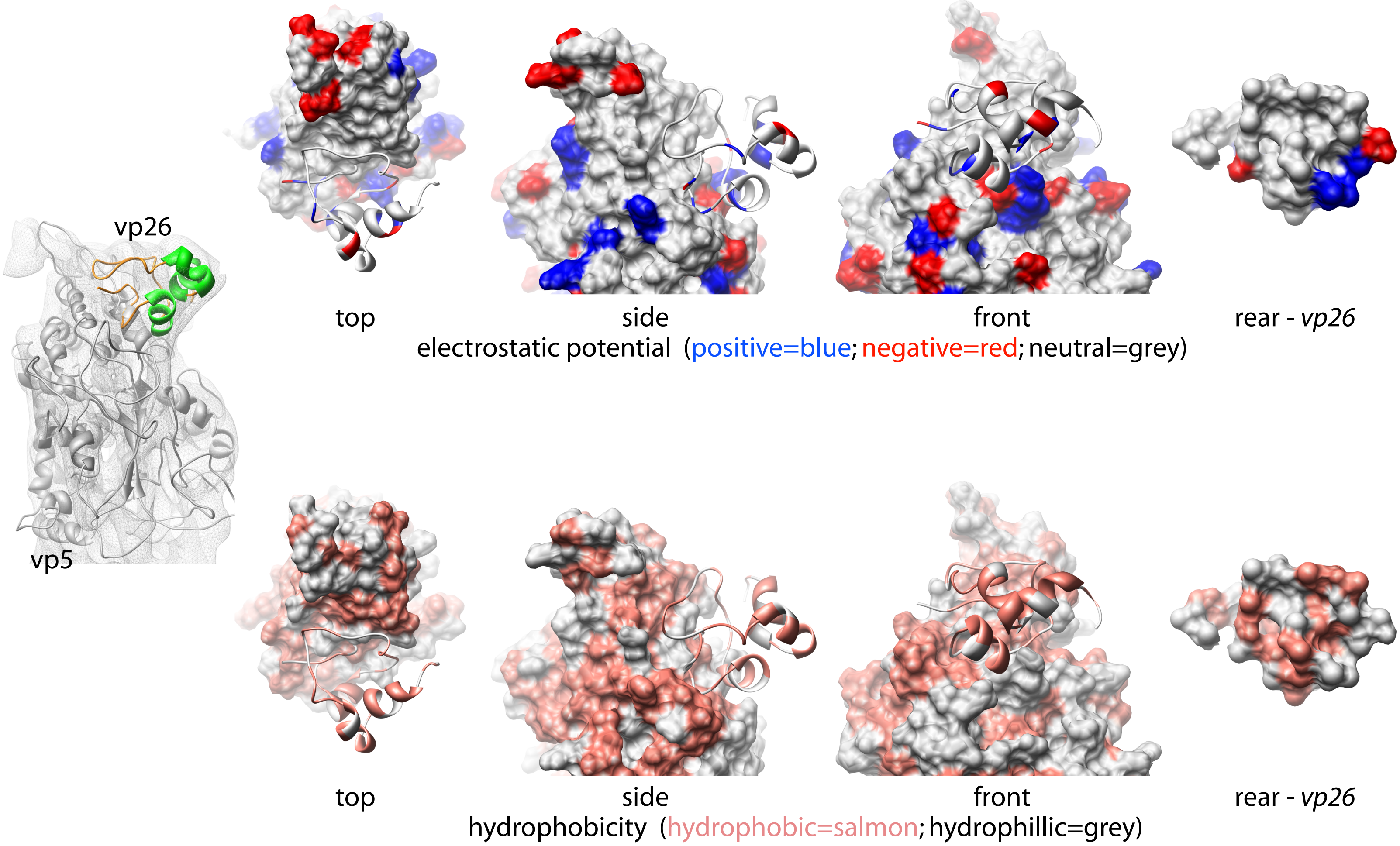
vp26
vp5
top
side
front
rear - vp26
electrostatic potential (positive=blue; negative=red; neutral=grey)
top
side
front
rear - vp26
hydrophobicity (hydrophobic=salmon; hydrophillic=grey)
